# Enhanced excitability and suppression of A-type K^+^ currents in joint sensory neurons in a murine model of antigen-induced arthritis

**DOI:** 10.1038/srep28899

**Published:** 2016-07-01

**Authors:** Lintao Qu, Michael J. Caterina

**Affiliations:** 1 Department of Neurosurgery, Neurosurgery Pain Research Institute, Johns Hopkins University School of Medicine, Baltimore, MD, 21205, USA; 2 Department of Biological Chemistry, Johns Hopkins University School of Medicine, Baltimore, MD, 21205, USA; 3 Solomon H. Snyder Department of Neuroscience, Johns Hopkins University School of Medicine, Baltimore, MD, 21205, USA.

## Abstract

Pain is a dominant symptom of rheumatoid arthritis (RA) and its adequate treatment represents a major unmet need. However, the cellular mechanisms that drive arthritis pain are largely unexplored. Here, we examined the changes in the activity of joint sensory neurons and the associated ionic mechanisms using an animal model of antigen-induced arthritis (AIA). Methylated-bovine serum albumin (mBSA), but not vehicle challenge, in the ankle of previously immunized mice produced time-dependent symptoms of arthritis, including joint inflammation, primary mechanical hyperalgesia in the ipsilateral ankle, and secondary mechanical and heat hyperalgesia in the ipsilateral hindpaw. *In vivo* electrophysiological recordings revealed that Dil-labeled joint sensory neurons in AIA mice exhibited a greater incidence of spontaneous activity, mechanically evoked after-discharges, and/or increased responses to mechanical stimulation of their receptive fields, compared to control animals. Whole-cell recordings *in vitro* showed that AIA enhanced the excitability of joint sensory neurons. These signs of neuronal hyperexcitability were associated with a significant reduction in the density of A-type K^+^ currents. Thus, our data suggest that neuronal hyperexcitability, brought about in part by reduced A-type K^+^ currents, may contribute to pain-related behaviors that accompany antigen-induced arthritis and/or other antigen-mediated diseases.

Rheumatoid arthritis (RA) is a common chronic autoimmune disease characterized by bone destruction and joint inflammation^1^. Joint pain is a predominant clinical feature of RA and represents a significant health burden^2^,^3^. However, the underlying mechanisms that drive arthritis pain are largely unexplored.

Antigen–induced arthritis (AIA) is one of the most extensively used animal models for studies of mechanisms underlying RA-induced pain^4^,^5^. It is an easily reproducible and translational immunization model in which arthritis is induced by exogenous antigens, such as ovalbumin^6^ or methylated bovine serum albumin (mBSA)^7^,^8^. Compared with arthritis models induced by Complete Freund’s Adjuvant (CFA) or carrageenan, the AIA model is driven by both the innate and adaptive immune systems and in this way better mimics some of the major histological and immunological features of human RA, including joint swelling, bone destruction and hypernociception^4^. In addition, unlike collagen-induced arthritis, collagen antibody-induced arthritis or the K/BxN serum transfer model, the AIA model generates RA-like pathology only in one joint, which facilitates the evaluation of pain-like behaviors^4^. Thus, this model offers certain advantages for the studies of RA-associated pain.

The joint structure is richly innervated by nociceptive fibers (Aδ and C) that are likely to be the sources of painful inputs to the spinal cord during RA^9^. Enhanced excitability of joint nociceptors is thought to contribute to the ongoing pain and hyperalgesia that accompany this disorder and other immune-related diseases in humans. Several studies have documented the effects of proinflammatory cytokines (e.g., interleukin (IL)-1β, IL-6, and IL-17) on arthritis-associated pain behaviors, on the sensitivity of teased peripheral joint afferents to joint rotation in the AIA model, or on the excitability of cultured dissociated joint sensory neurons^10^,^11^,^12^,^13^,^14^. In some cases, these studies have also described associated changes in the expression of cytokine receptors and transduction channels^11^,^14^,^15^. Moreover, spontaneous activity (SA) and increased mechanical sensitivity were observed in joint sensory afferents in a rat model of osteoarthritis, and were implicated in the maintenance of osteoarthritis pain^16^,^17^. However, to our knowledge, there have been no studies addressing the possible existence of such spontaneous activity or of pathological after-discharges in intact individual joint sensory neurons in models of AIA. Thus, much remains to be learned about the neurophysiological changes associated with joint pain in AIA.

Voltage-gated K^+^ (Kv) channels are widely expressed in primary sensory neurons and play critical roles in the regulation of neuronal excitability. Kv currents recorded from dorsal root ganglion (DRG) neurons consists of two major subtypes, transient A-type K^+^ currents (I_A_) and sustained delayed rectifier K^+^ currents (I_K_), both of which have been implicated in the generation of pain sensation^18^. Knockdown of A-type K^+^ channel expression in primary sensory neurons induced mechanical hyperalgesia^19^. In addition, there is increasing evidence that the expression and activity of Kv channels in primary sensory neurons are downregulated under inflammatory and neuropathic pain conditions and that these channels are involved in the maintenance of a chronic pain state^20^,^21^,^22^. Consistent with this notion, CFA- induced joint inflammation produced downregulation of A-type K^+^ channels in joint-innervating sensory neurons, possibly contributing to mechanical allodynia in the inflamed joint^23^,^24^. However, whether similar changes also occur in joint sensory neurons in the context of AIA remains unknown.

In the present study, we employed both *in vitro* and *in vivo* approaches to examine changes in the excitability of joint sensory neurons in a murine AIA model induced by mBSA. Moreover, we specifically investigated the possibility of alterations in K^+^ channels in joint sensory neurons in this model.

## Results

### mBSA challenge induced arthritis accompanied by pain-like behaviors

**Figure 1:**
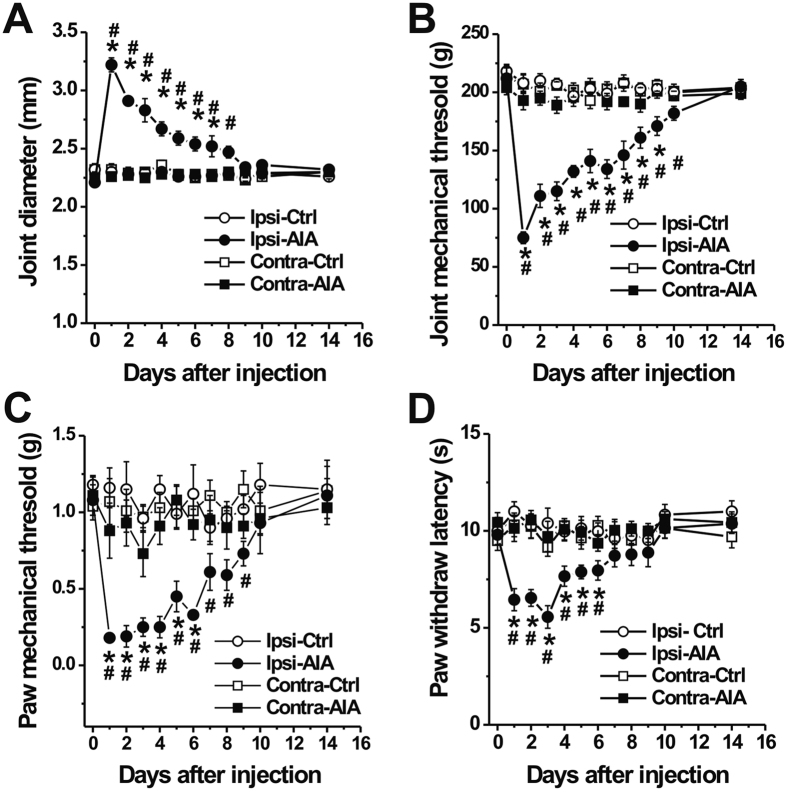
mBSA challenge induces joint swelling and pain-like behaviors in previously immunized mice. Antigen-induced arthritis (AIA) was produced in the right ankle. (**A**) Ankle diameter was significantly increased one day after mBSA (30 μg in 10 μl saline) was injected into the ankle of immunized mice (Ipsi-AIA, n = 12–13 mice), whereas injection of saline alone (vehicle) had no significant effects (Ipsi-Ctrl, n = 13 mice). There were no significant changes in joint diameter on the contralateral (Contra) side. (**B**) Primary mechanical withdrawal thresholds were evaluated when the ankle joint was compressed using calibrated electronic forceps. Withdrawal threshold for mechanical stimulation of the ankle joint was significantly lower in mBSA-challenged mice (n = 12–13 mice) compared to control animals (n = 13 mice). (**C,D**) Mice with AIA exhibited lower paw withdrawal thresholds for mechanical stimulation of glabrous paw skin with von-Frey filaments (**C**) n = 8 mice for each group), and shorter paw withdrawal latencies for noxious heat stimulation of the ipsilateral glabrous paw skin compared to controls (**D**) control: n = 13 mice; AIA: n = 12–13 mice). There were no significant differences between groups in joint mechanical threshold or in paw skin mechanical or heat thresholds on the contralateral side. *p < 0.01, AIA versus control; ^#^p < 0.01 versus baseline at day 0, Two-way ANOVA with repeated measures followed by Bonferroni adjustments.

Intraarticular (i.a.) injection of mBSA to the ankle joint of mice that had previously been systemically sensitized to this antigen induced progressive arthritis, characterized by strong joint swelling, consistent with previous findings with mBSA and other antigens^8^,^13^. Ankle diameter was significantly increased on day 1 after mBSA challenge as compared with saline-treated controls (Fig. 1A, p < 0.01, two-way ANOVA with Bonferroni post hoc test). In addition, mice with AIA exhibited pronounced mechanical hyperalgesia to pressure applied to the inflamed ankle, as compared to control animals (Fig. 1B; p < 0.01; two-way ANOVA with Bonferroni post hoc test). The compression-withdrawal thresholds decreased to a nadir 1 day after AIA induction and gradually returned to control levels by day 14. However, no significant changes in mechanical threshold were observed in the contralateral ankle after AIA (p > 0.05, two-way ANOVA). Compared with control animals, AIA in the ankle also lowered the threshold for responsiveness to punctate mechanical stimulation in the glabrous skin of the ipsilateral hindpaw, indicative of secondary mechanical hyperalgesia remote from the inflamed site (Fig. 1C; p < 0.01, two-way ANOVA with Bonferroni post hoc test). No such significant changes occurred in the contralateral hindpaw after AIA (p > 0.05, two-way ANOVA). In addition, withdrawal latency to noxious heat in the glabrous hindpaw skin was reduced ipsilaterally one day after mBSA challenge and was fully resolved by day 10 (Fig. 1D; p < 0.05, two-way ANOVA with Bonferroni post hoc test). The paw thermal hyperalgesia was not observed in either hindpaw of control mice and did not extend to the contralateral hindpaw of AIA mice (p > 0.05, two-way ANOVA).

### mBSA challenge produced spontaneous activity in a portion of joint-innervating DRG neurons *in vivo*

**Figure 2:**
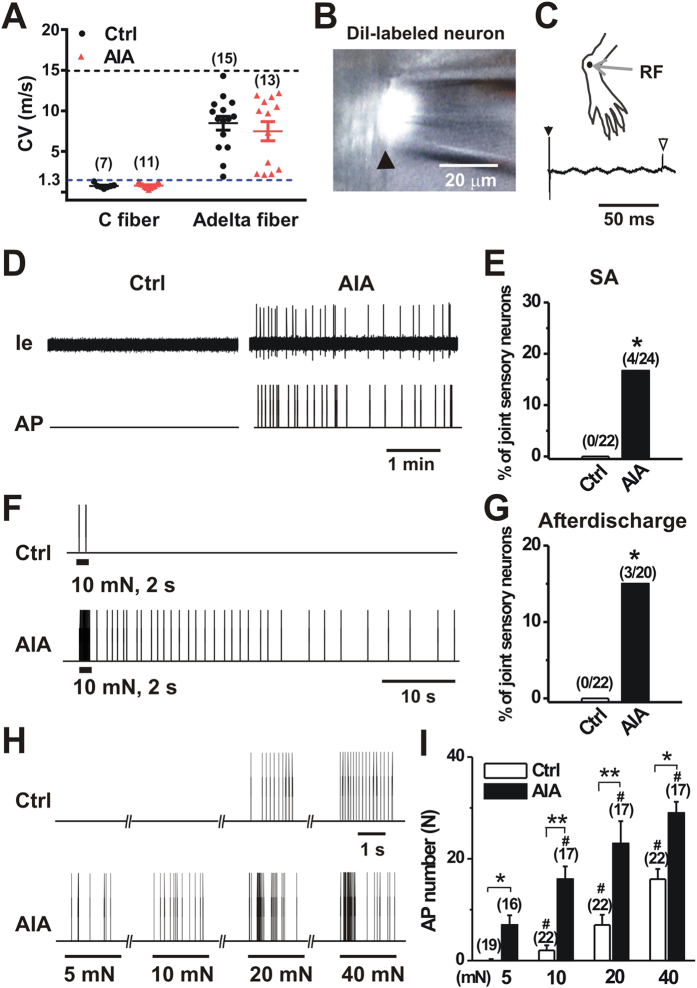
AIA produces spontaneous activity and enhanced mechanical sensitivity in joint sensory neurons. (**A**), Distribution of the recorded C and Aδ fibers innervating the ankle of control and AIA mice. C and Aδ fibers had conduction velocities (CVs) < 1.3 m/s (below blue line) and 1.3–15 m/s (between blue and dark line), respectively. There were no significant differences in mean CVs of joint afferents between control and AIA mice. p > 0.05 versus control, unpaired t-test. (**B**) Cell body of a DiI fluorescent neuron (arrow) in control mouse suctioned against the tip of a recording pipette. (**C**) Top, Receptive field (RF) of joint sensory neuron shown in (**B**). Bottom, action potential (AP; open arrow) evoked by electrical stimulation (arrow) to the RF revealed a CV of 0.41 m/s. (**D**) Representative extracellular current (Ie) traces and AP markers indicate the presence of abnormal spontaneous activity (SA) in joint sensory neurons of AIA mice but not those of control animals. (**E**) Prevalence of SA in AIA versus controls. *p < 0.05, Fisher’s exact test. (**F**) Responses of joint sensory neurons in control and AIA mice to a 2 s, 10 mN mechanical stimulus delivered via a 100 μm probe. The neurons innervating mBSA-challenged ankle were initially silent but showed prolonged after-discharges following mechanical stimulation. No mechanically evoked after-discharges occurred in joint sensory neurons of control animals. (**G**) Prevalence of mechanically evoked after-discharges in joint sensory neurons of AIA mice versus controls. * p < 0.05, Fisher’s exact test. (**H**) Representative responses of joint sensory neurons of control and AIA mice to mechanical stimulation of their RF with von Frey filaments (100 μm tip diameter) at the indicated bending forces. (**I**) The mean number of APs evoked by mechanical stimuli 2 s in duration was greater in joint sensory neurons of AIA mice compared to those of controls. *p < 0.01, **p < 0.001 versus control, ^#^p < 0.01 versus 5 mN, two-way ANOVA with repeated measures followed by Bonferroni adjustments. In all panels, numbers of neurons tested are in parentheses.

Joint pain may result from the activation and sensitization of nociceptive nerve fibers that supply the joint. We therefore asked whether joint-innervating DRG neurons became hyperexcitable after AIA. To address this issue in a physiologically relevant setting, we employed an *in-vivo* recording preparation in which the activity in the somata of individual sensory neurons in DRG can be interrogated with preservation of both peripheral and spinal connections^25^. Following retrograde labeling of joint innervating sensory neurons with DiI, extracellular electrophysiological recordings were obtained from Dil-labeled mechanosensitive sensory neurons with a RF within the vehicle- or mBSA-treated ankle (Fig. 2A–C). A total of 22 and 24 joint sensory neurons were recorded on day 1 after vehicle and mBSA challenge, respectively. All the neurons tested had CVs within the ranges of C-(≤1.3 m/s) or Aδ-fibers (1.3–15 m/s). The mean CVs of C- or Aδ- fibers were similar between control and AIA mice (Fig. 2A; p > 0.05, unpaired t-test). In control mice, all joint sensory neurons tested (including 7 C- and 15 Aδ- fibers) were silent in the absence of exogenous stimuli, with no detectable SA (Fig. 2D). In contrast, 4 of 24 (16.7%) joint sensory neurons in AIA mice (including 11C- and 13 Aδ- fibers) exhibited SA (Fig. 2D). All these 4 neurons with SA were C- fibers. The proportion of spontaneously firing neurons was significantly greater in AIA mice compared to control animals (Fig. 2E; p < 0.05, Fisher’s exact test).

Since mechanical sensitization of joint sensory neurons has been proposed be a critical neuronal mechanism of mechanical hyperalgesia, we next examined whether the responses of joint sensory neurons to mechanical stimuli were enhanced after AIA. To avoid the confounding of spontaneous firing, we exclusively focused on joint sensory afferents that did not exhibit SA. Three of 20 (15.0%) joint sensory neurons in AIA mice displayed abnormal after-discharges in response to punctate mechanical stimulation of their RF. Of these 3 neurons, 2 were C- fibers while the remaining one was Aδ- fiber. In contrast, no mechanically evoked after-discharges occurred in 22 joint sensory neurons recorded in control mice (Fig. 2F). The proportion of joint sensory neurons with abnormal mechanically evoked after-discharges was significantly larger in AIA mice as compared to control animals (Fig. 2G; p < 0.05, Fisher’s exact test). Overall, 7 of 24 joint afferents in AIA mice exhibited abnormal activity (SA and mechanically evoked after-discharges) whereas it was not observed in all 22 joint afferents tested in control animals (p < 0.05; Fisher’s exact test). In addition, we compared the mechanical sensitivities of joint sensory neurons that did not exhibit either SA or after-discharges for two groups (Fig. 2H). The mean number of APs evoked by each mechanical force (5 mN to 40 mN) was significantly greater in joint sensory neurons of AIA mice than those of controls (Fig. 2I; p < 0.01, two-way ANOVA with Bonferroni post hoc test). These results indicate that the excitability and mechanical sensitivity of the peripheral terminals of a portion of joint sensory neurons were enhanced by AIA.

### mBSA challenge increased the excitability of dissociated joint-innervating DRG neurons

**Figure 3:**
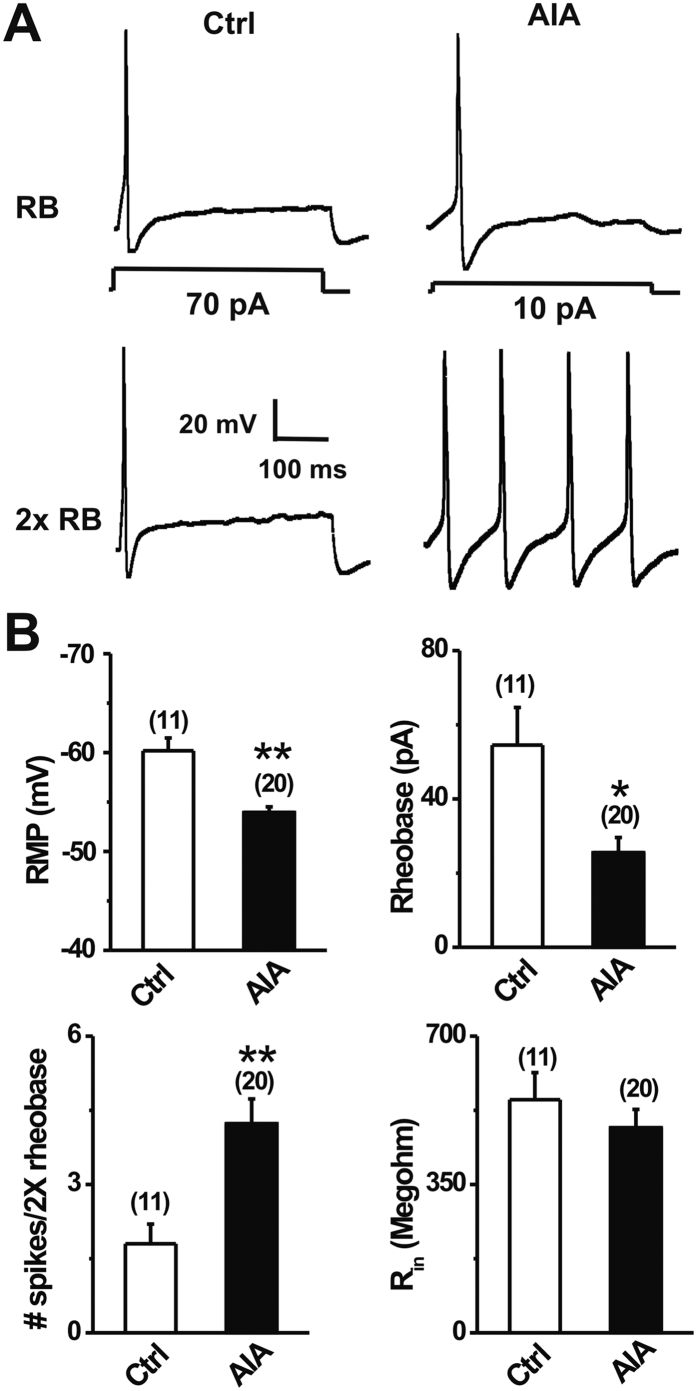
mBSA challenge increases the excitability of joint sensory neurons. (**A**) Representative traces of APs elicited at rheobase and twice rheobase in Dil-labeled joint sensory neurons from control (Ctrl) and AIA mice. mBSA challenge caused a reduction in rheobase while increasing the number of APs evoked by a 2x rheobase current injection. (**B**) Joint sensory neurons from AIA mice exhibited a more depolarized resting membrane potential (RMP), lower mean rheobase, and greater number of action potentials at 2X rheobase, as compared to the control group. No significant difference in input resistance (R_in_) was observed between groups. Cell numbers tested are indicated in parentheses. *p < 0.05, **p < 0.001 versus control animals, unpaired t-test.

To further investigate the effects of AIA on the excitability of joint nociceptors, whole-cell recordings were made from acutely dissociated, retrogradely DiI-labeled DRG neurons that supply the ankle joint 1 day after i.a injection of mBSA (AIA) or saline (control) (Fig. 3). In comparison with controls, joint sensory neurons from AIA mice exhibited significantly more depolarized resting membrane potentials (Fig. 3; p < 0.001, unpaired t-test). The average rheobase of joint sensory neurons from AIA mice was markedly lower than that in control animals (Fig. 3, p < 0.05, unpaired t-test). In addition, the numbers of APs evoked at twice rheobase were significantly greater in joint sensory neurons from AIA mice (Fig. 3; p < 0.001; unpaired t-test). In contrast, no significant differences were observed in input resistance (Fig. 3) or cell capacitance (control: 16.9 ± 0.8 pF, n = 11; AIA: 17.6 ± 1.3 pF, n = 20) of joint sensory neurons from the two groups (p > 0.05, unpaired t-test).

### mBSA challenge reduced voltage-gated K^+^ currents

**Figure 4:**
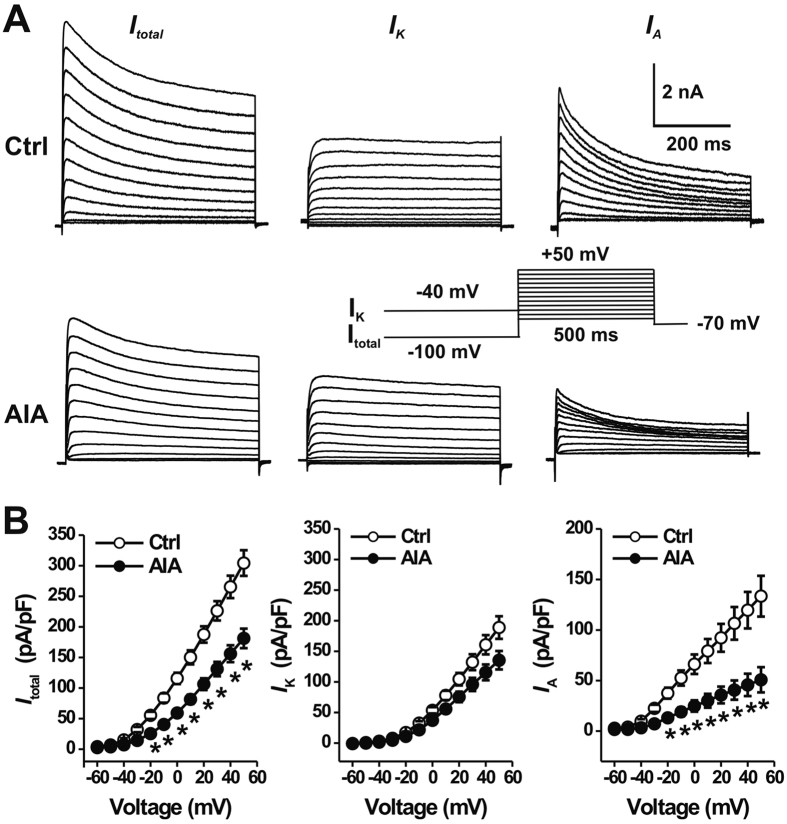
AIA reduces the I_A_ density in joint sensory neurons. Join innervating DRG neurons were retrogradely labeled with Dil. (**A**) Representative traces of voltage-gated K^+^ currents in two joint sensory neurons, one from a control (Ctrl) mouse (top) and the other from a AIA mouse (bottom). Total K^+^ currents (I_total_; left) were elicited by s series of 500-ms test pulses from −60 to +50 mV in 10-mV steps, preceded by a 500-ms prepulse of −100 mV (inset). Delayed rectifier K^+^ (I_K_) currents (middle) were generated using the same series of test pulses but preceded by a 500-ms prepulse of −40 mV. A-type K^+^ (I_A_) currents (right) were obtained by digitally subtracting I_K_ from I_total_. (**B**) The peak current densities of I_total_ (left) and I_A_ (right) were significantly smaller in AIA mice (closed circles, n = 16 neurons), as compared to controls (open circles, n = 18 neurons). There were no significant differences in I_K_ density (middle) between two groups. *p < 0.001 versus control, one-way repeated measures analysis of variance with Tukey post hoc comparisons.

Voltage-gated K^+^ (Kv) channels are crucial for controlling neuronal excitability by regulating AP threshold and firing rate^26^. We therefore next examined whether the increased neuronal excitability in AIA mice was due to alterations in Kv currents. Command potentials delivered after a holding potential of −100 mV were used to evoke a total voltage-activated K current (I_total_), which included both I_K_ and I_A_ (Fig. 4A). A holding potential at −40 mV was used to inactivate I_A_ and thereby isolate I_K_. Subsequent subtraction of the current traces evoked by the two holding potentials then allowed us to calculate I_A_ (Fig. 4A). The mean peak current densities of I_total_ and I_A_ were significantly lower in joint sensory neurons from AIA mice (n = 13) compared to those in neurons from control animals (n = 16) (Fig. 4B; p < 0.01, repeated one-way ANOVA with Bonferroni post hoc test). In contrast, there were no significant differences in I_K_ density between the two treatment groups (Fig. 4B; p > 0.05, repeated one-way ANOVA).

**Figure 5:**
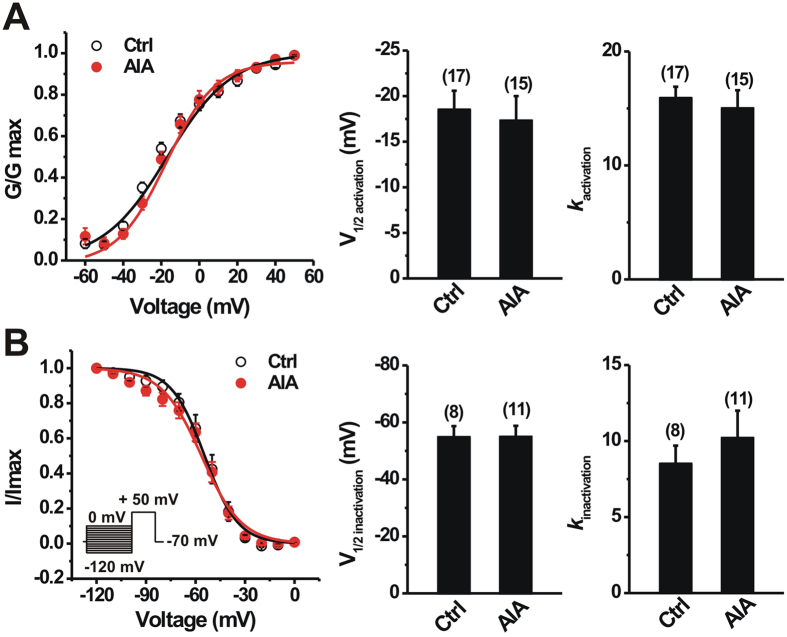
Effects of AIA on voltage-dependent activation and steady-state inactivation of I_A_ in joint sensory neurons. (**A**) For activation curves, normalized conductance (G/Gmax) was plotted against test pulse voltage and fitted to a Boltzmann function. AIA (closed circles) did not alter activation curve of I_A_ compared with the control (Ctrl; open circles) in joint sensory neurons. Bar graph shows that there were no significant changes in V1/2 or k for I_A_ activation between two groups. (**B**) For inactivation curves, a long (500 ms) conditional step of various voltages from −120 mV to 0 mV in 10 mV increment was followed by a testing pulse (300 ms) to +50 mV (inset). Normalized current (I/Imax) was plotted against conditional step potentials and fitted to a negative Boltzmann function. No changes in the inactivation curve of I_A_ in joint sensory neurons occurred after AIA (closed circles) compared to control (Ctrl; open circles) mice. Bar graph showed that there were no significant differences in V1/2 or k for I_A_ inactivation between control and AIA mice. Numbers of neurons tested are in parentheses (**A,B**). p > 0.05 versus control, unpaired t-test.

We further tested whether the reduction in I_A_ density observed in AIA group was due to significant alterations in the voltage-dependence of activation or inactivation of K^+^ currents. The voltage-dependence of I_A_ activation was not significantly different between control and AIA groups (Fig. 5A; p > 0.05, unpaired t-test). The mean activation midpoint (V_1/2act_) of I_A_ did not significantly differ between the two groups (Fig. 5A; p > 0.05, unpaired t-test). There also was no significant difference in the mean slope factor for I_A_ activation (Fig. 5A; p > 0.05, unpaired t-test). In addition, mBSA challenge did not significantly affect the voltage-dependence of steady-state inactivation of I_A_ (Fig. 5B; p > 0.05; unpaired t-test). No significant differences were observed between control and AIA groups in the mean values of V_1/2 inact_ or the slope factor for I_A_ current inactivation (Fig. 5B; p > 0.05, unpaired t-test).

## Discussion

Although the inflammatory component of AIA has received considerable attention, little is known of the effects of AIA on the functional properties of primary sensory neurons that innervate the joint. Our study specifically focused on pain-related behaviors and changes in the excitability of joint sensory neurons following AIA. We provide several observations that support the presence of enhanced excitability of these neurons during AIA. First, *in vivo* electrophysiological recordings revealed that a proportion of DRG neurons innervating the mBSA-challenged ankle joint, but not those in control animals, became spontaneously active. SA in nociceptors has also been reported in models of joint and cutaneous inflammation, and peripheral nerve injury^16^,^25^,^27^,^28^,^29^, but this is to our knowledge the first demonstration of SA in the AIA model. Second, a subset of sensory neurons with their receptive fields in the inflamed ankle exhibited abnormal mechanically evoked after-discharges. Third, even among neurons without SA or after-discharges, a greater mean number of mechanically evoked APs were observed in joint sensory neurons after mBSA challenge, indicating mechanical hypersensitivity these neurons in the context of AIA. Fourth, the dissociated cell bodies of joint sensory neurons became hyperexcitable under arthritic conditions, as indicated by a depolarized resting membrane potential, a significant decrease in rheobase and an increase in the number of action potential discharges evoked at twice rheobase.

Collectively, our findings both reinforce and extend those of previous studies on the mechanisms underlying joint pain in AIA. Furthermore, whereas in prior studies, neuronal activity was measured from teased peripheral joint nerve fibers^10^,^11^,^16^,^30^, our present study included *in vivo* recordings from the somata of anatomically intact, retrogradely labeled DRG neurons innervating the ankle. These findings thus lay the foundation for future studies in which this assay can be combined with genetic labeling^25^ and optogenetic methods to study nociceptive processing in specific subsets of joint sensory neurons.

In this study, we characterized the mechanical sensitivity of joint sensory afferents using von-Frey filaments rather than joint rotation, since there are no commercially available devices to produce consistent and quantifiable angles and forces of ankle joint rotation in the mouse. The kinetics of force delivery in our study might have been subject to small variations upon repetition because von-Frey stimuli were applied by hand. However, such punctate mechanical stimuli have been commonly used to determine mechanical sensitivity of joint sensory neurons under various pathologic conditions^16^,^31^,^32^. In addition, we confined our recordings to DRG neurons that had been retrogradely labeled by DiI injected into the ankle joint. For our *in vivo* recordings, this enrichment strategy was coupled with restriction to neurons with mechanical RFs on the ankle joint. Although the precise anatomical structures innervated by these sensory neurons were not identified in the present study, it is likely that they had at least one branch projecting to the joint and that they innervated the tissues immediately beneath the site of von Frey filament stimulation.

Kv channels play critical roles in controlling neuronal excitability by determining RMP and affecting rheobase and spike frequency^33^,^34^. Our findings demonstrated that AIA caused a significant decrease in I_A_ magnitude. This change in I_A_ may account in part for the hyperexcitability of joint sensory neurons in the context of AIA. Possible mechanisms for reduced I_A_ density include changes in channels properties and down-regulation of I_A_ channel expression in joint sensory neurons. Since we observed no significant alterations in the kinetics of activation or inactivation of I_A_ in joint sensory neurons from AIA mice, it appears more likely that the suppression of I_A_ was due to the down-regulation of I_A_ channel expression. In DRG neurons, five subtypes of I_A_ channels have been identified: Kv1.4, Kv3.4, Kv4.1, Kv4.2 and Kv.4.3^19^,^35^,^36^. Among these, the Kv1.4 channel is the dominant I_A_ subunit expressed in nociceptors^36^. Further investigations are required to determine the molecular basis of changes in I_A_ produced by AIA. Reductions in I_A_ have been proposed to underlie increased neuronal excitability in other models of peripheral inflammation. For example, joint inflammation induced by CFA caused a decrease in both I_A_ magnitude and the expression of Kv 1.4 in small-diameter trigeminal ganglion neurons^23^,^24^. The mechanisms whereby peripheral inflammation reduces the activity and/or expression of I_A_ channels require further investigation. The inflammatory milieu surrounding primary joint afferent fiber terminals in the setting of AIA contains numerous inflammatory mediators^37^, such as IL-1β and glial cell line-derived neurotrophic factor (GDNF). The latter may be retrogradely transported to cell bodies of DRG neurons and contribute to the reduced I_A_ observed in present study. Indeed, both GDNF and IL-1β have been demonstrated to reduce I_A_ in nociceptors^38^,^39^. In addition, autoantibodies against neuronal Kv channels have been detected in certain autoimmune diseases, and might decrease the activity of Kv channels to produce neuronal hyperexcitability^40^. Although the decrease of I_A_ we observed likely contributes to enhanced excitability in AIA, we cannot rule out the involvement of other ion channels and/or inflammatory mediators. Since no significant changes in input resistance of joint sensory neurons occurred during AIA, it is unlikely that leak channels are involved in the observed neuronal hyperexcitability.

AIA is a reproducible model that replicates many features of joint pathology and associated pain symptoms observed in human RA^4^. Surprisingly, few studies have assessed primary mechanical hyperalgesia in the joints of mice with AIA, although both primary and secondary hypernociception were observed in the rats with AIA^11^,^13^,^15^. In this study, we showed that challenging previously immunized mice with mBSA induced primary mechanical hyperalgesia in the ankle, that lasted for approximately 1 week, a time course similar to that of joint swelling. Consistent with previous studies^8^,^15^, AIA mice exhibited secondary mechanical and heat hyperalgesia in the ipsilateral hindpaw. Secondary hyperalgesia is thought to arise from central sensitization, a state in which a given input to the spinal cord results in a larger relative pain response. In some but not all previous studies, secondary mechanical hypersensitivity also developed in the contralateral hindpaw^5^,^41^. However, contralateral effects were not observed in our study. There are several possible relationships among the neurophysiological and behavioral AIA-associated changes described in this study. The increased excitability recorded in the cell bodies of joint sensory neurons from AIA mice *in vitro* may be linked to the hyperexcitability of their peripheral terminals *in vivo*. This hyperexcitability, in turn, could account for the abnormal mechanically evoked after-discharges and/or mechanical hypersensitivity observed when their RFs were mechanically stimulated *in vivo*. Increased mechanical sensitivity of joint afferents themselves might reasonably be expected to contribute to the behavioral signs of primary mechanical hyperalgesia and allodynia. In addition, however, SA and mechanically evoked after-discharges might not only provide nociceptive inputs that trigger pain perception, but might also contribute to the establishment and maintenance of central sensitization. Previous studies have demonstrated the existence of spinal hyperexcitability following knee joint inflammation, and provided evidence that proinflammatory cytokines such as tumor necrosis factor α (TNFα) contribute to such sensitization^42^. SA in nociceptors has been shown to induce and/or maintain central sensitization^43^,^44^. Moreover, inputs from joint and muscle nociceptors may produce a longer-lasting central sensitization than input from cutaneous nociceptors^45^,^46^. Coupled with the additional action potentials of mechanically-associated after-discharges, SA in the AIA model might therefore contribute to pain by multiple mechanisms.

In conclusion, our results demonstrate that AIA causes neuronal hyperexcitability and suppression of I_A_ in joint sensory neurons that might contribute to joint pain associated with AIA. These findings may suggest new strategies for the treatment of pain accompanying AIA as well as other antigen-mediated disorders. Since no single experimental model of arthritis recapitulates all aspects of human RA^4^, however, further experiments will be needed to understand which of these neurophysiological changes are common across models.

## Methods

### Animals

C57BL/6 male mice used in the study were 2 to 3 months of age and weighed 20–30 g and were originally obtained from Jackson Laboratories (Bar Harbor, ME). All experimental procedures were approved by the Institutional Animal Care and Use Committee of Johns Hopkins University School of Medicine and were in accordance with the guidelines provided by the National Institute of Health and the International Association for the Study of Pain.

### Model of antigen-induced arthritis (AIA)

The antigen, mBSA (Sigma, St. Louis, MO), was used to elicit arthritis in the mouse as a model of human RA, as described previously^8^,^13^. Briefly, Mice were sensitized with 500 μg of mBSA in 200 μl of an emulsion containing 100 μl saline and 100 μl CFA (1 mg/ml) and delivered by subcutaneous (s.c.) injection to the caudal back skin with a sterile syringe and 25G needle. The mice were boosted with the same preparations on day 7. Immunized mice were challenged on day 21 by i.a. injection of mBSA (30 μg; 10 μl in saline) or saline alone (vehicle) to the right ankle of the hindlimb. Ankle diameter was measured with digital calipers before and after the induction of AIA as an indicator of joint inflammation.

### Behavioral testing

All the behavioral tests of the mBSA immunized mice were performed on day 0 (immediately before mBSA challenge) and up to day 14 following i.a injection of mBSA or saline vehicle. Since ankle joint inflammation was obvious during the course of AIA, the behavioral tester could not be blinded to the treatments. Primary mechanical hyperalgesia in both ankle joints was measured by applying ascending forces to the ankle with electronic blunt forceps (IITC Inc., Woodland Hills, CA). The cutoff force was set at 350 g to avoid joint damage. The mechanical threshold was defined as the force at which the mouse withdrew its hindlimb forcefully or vocalized^11^,^47^. The mechanical threshold in the joint was averaged over three measurements obtained at intervals of at least 5 min. Secondary mechanical hyperalgesia in the glabrous skin of both paws was evaluated using the up-down von Frey filament assay^48^. Secondary thermal hyperalgesia in the glabrous hind paw skin was assessed by measuring withdrawal latency to noxious heat stimuli delivered using a radiant heat source^49^. Heat response latencies were averaged over three measurements obtained at intervals of at least 3 min.

### Retrograde labeling of ankle joint afferents

For *in vivo* and vitro studies, DRG cell bodies with their afferent fibers innervating vehicle (saline)- or mBSA-treated ankle joints were identified by the presence of a retrogradely transported red fluorescent dye, DiI (Sigma, St. Louis, MO). Dil injected into the right ankle (2.5 mg /ml, 10 μl in 25% ethanol) at least 1 week prior to i.a. injection of mBSA or saline.

### *In- vivo* electrophysiological recordings

The properties of DRG neurons innervating the hind limb ankles of mice were recorded *in vivo* on day 1 after the induction of AIA using extracellular recording as described^25^. Briefly, under isoflurane anesthesia delivered via intratracheal ventilation (SomnoSuite, Kent Scientific Corp., Torrington, CT), the lumbar spinal column was exposed and a laminectomy performed at the L2-L6 levels. The L4 or L5 DRG was exposed and superfused with warm (~37 °C) oxygenated artificial cerebrospinal fluid (ACSF) at flow rate of 3 ml/min within a pool formed by a ring to which the skin was sewn. The ACSF contained (in mM): 130 NaCl, 3.5 KCl, 24 NaHCO_3_, 1.25 NaH_2_PO_4_, 1.2 MgCl_2_, 1.2 CaCl_2_, and 10 dextrose. The solution was bubbled with 95% O_2_ and 5% CO_2_ and had a pH of 7.4 and an osmolarity of 290~310 mOsm. After removal of the epineurium, the neurons on the surface of the DRG were viewed by reflection microscopy on a Nikon FN1 upright microscope equipped with a mercury light source (Nikon, Mellvile, NY) and an infrared camera (DAGE-MTI, Michigan City, IN). Epifluorescence imaging was also used to identify DiI-labeled joint-innervating neurons.

Extracellular recordings were made on individual DRG cell bodies using a polished suction micropipette electrode with a tip of 20–30 μm. Pipettes were pulled from borosilicate glass capillaries (Sutter Instruments; Novato, CA) using a P97 micropipette puller(Sutter Instruments). The occurrence of action potentials (APs) was recorded extracellularly using a Multiclamp 700B amplifier and pCLAMP10 software (Molecular Device, Sunnyvale, CA). The peripheral receptive field (RF) of an individual joint-innervating DRG neuron was identified by probing the skin over and around the exposed ankle with a hand-held blunt glass probe. Only DRG neurons that had a mechanical RF in the ankle were included. The mechanical sensitivity of joint sensory afferents was assessed by poking their RF with a set of calibrated von Frey monofilaments with a fixed tip diameter (100 μm). Each monofilament was applied for 2 s with an inter-stimulus interval of 3 min. Mechanical responses were quantified as the mean number of evoked APs during 2 s mechanical stimulation^16^. Conduction velocity (CV) was obtained by electrically stimulating the RF with two wire electrodes and calculated by dividing the conduction distance between the stimulation electrode and the soma of the recorded neurons by the latency to a spike peak. The fibers with CV of 1.3 m/s or less were classified as C-fibers whereas those with CV between 1.3 and 15 m/s were classified as Aδ-fibers^30^. In this study, C fiber and Aδ fibers were preferentially studied. A neuron was classified as spontaneously active only if spontaneous ongoing discharges occurred during a 3 min period without any external stimulation^50^.

### Culture of dissociated DRG neurons

On day 1 after the induction of AIA, L4-L5 lumbar DRGs, ipsilateral to either the saline- or mBSA-treated ankle, were harvested and placed in oxygenated complete saline solution (CSS) for cleaning and then mincing^25^,^51^. CSS consisted of (in mM): 137 NaCl, 5.3 KCl, 1 MgCl_2_, 3 CaCl_2_, 25 Sorbitol, and 10 HEPES, adjusted to pH 7.2 with NaOH. For 20 min the DRGs were digested with 0.35 U/ml of Liberase TM (Roche Diagnostics Corp., Indianapolis, IN) and then for 15 min with 25 U/ml of Liberase TL (0.25 U/ml; Roche Diagnostics Corp.) and papain (30 U/ml, Worthington Biochemical, Lakewood, NJ) in CSS containing 0.5 mM EDTA at 37 °C. The tissue was triturated with a fire-polished Pasteur pipette. The DRG neurons were suspended in DMEM medium containing 1 mg/ml trypsin inhibitor and 1 mg/ml bovine serum albumin (Sigma) and then plated onto poly-D-lysine/laminin coated glass. The DMEM medium contained equivalent amounts of DMEM and F12 (Life Technologies Corp., Grand Island, NY) with 10% FCS (Sigma) and 1% penicillin and streptomycin (Invitrogen). The cells were maintained in 5% CO_2_ at 37 °C in a humidified incubator and used between 18–24 h after plating.

### Whole-cell patch clamp recordings from acutely cultured DRG neurons

Whole-cell recordings were made from small-diameter (≤25 μm) joint-innervating DRG neurons identified by the fluorescence of Dil using a Nikon TE200 inverted epifluorescence microscope. Electrophysiological recordings were performed at room temperature (20–22 °C) using a Axopatch 200B amplifier with pClamp 10 software (Molecular Device, Sunnyvale, CA), as described^52^,^53^. Signals were sampled at 10 kHz or 20 kHz and filtered at 2 kHz. Patch pipettes had a resistance of 3–4 MΩ. The series resistance was routinely compensated at 60–80%.

Resting membrane potential (RMP) was recorded for each neuron in current clamp mode after stabilization (within 3 min). A neuron was included only if the RMP was more negative than −40 mV and the spike overshoot was >15 mV. APs were evoked by a series of depolarizing current steps, each 500 ms duration, in increments of 50 pA up to 1 nA delivered through the recording electrode. The number of APs evoked by a suprathreshold stimulus was estimated by injecting a 500-ms depolarizing current of a magnitude at twice rheobase. Input resistance was obtained from the slope of a steady-state current-voltage plot in response to a series of hyperpolarizing currents steps from −200 to −50 pA in increments of 50 pA. For current clamp recordings, the internal solution contained (in mM): 120 K^+^-gluconate, 20 KCl, 1 CaCl_2_, 2 MgCl_2_, 11 EGTA, 10 HEPES-K^+^, 2 MgATP, with pH adjusted to 7.2 using Tris-base and osmolarity adjusted to 290–300 mOsm with sucrose. The external solution contained the following (in mM): 145 NaCl, 3 KCl, 2 MgCl_2_, 2 CaCl_2_, 10 glucose, and 10 HEPES, pH adjusted at 7.4 with NaOH. The liquid junction potential of 11 mV was corrected.

Kv currents were recorded in voltage clamp mode using the same internal solution as above, but the bath solution contained (in mM) 140 Choline Cl, 3 KCl, 1 CaCl_2_, 1 MgCl_2_, 0.1 CdCl_2_, 10 HEPES, and 10 glucose. The pH was adjusted to 7.4 with Tris base, and the osmolarity was adjusted to 300–310 mOsm^54^. I_A_ and I_K_ were separated biophysically by manipulating the holding potentials. Total K^+^ currents (I_total_) were evoked by a series of test pulses, each 500-ms in duration, from −60 mV to +50 mV in 10-mV steps, preceded by a 500-ms prepulse to −100 mV^54^. I_K_ was isolated by using a holding membrane potential of −40 mV. I_A_ was obtained by digitally subtracting the I_K_ component from I_total_. Linear leakage currents were digitally subtracted on-line using a P/4 procedure. To avoid the contamination of I_K_, steady-state inactivation of I_A_ was examined in presence of 25 mM TEA and elicited using a series of 500 ms prepulses from −120 to −10 mV with 10 mV increments, followed by a 300 ms test pulse of +60 mV^55^,^56^.

### Data analysis

Electrophysiological data were analyzed using pClamp 10 software and Origin 6.0 (OriginLab, Northampton, MA). For the analysis of voltage versus current relationships, the current density (pA/pF) was calculated by dividing the peak current by the cell capacitance. For the analysis of steady-state activation, K^+^ conductance (G) at each test pulse voltage (V) was calculated from the corresponding current (I) using the equation G = I/(V − V_rev_), where V_rev_ is the reversal potential of K^+^ current (−100 mV). Activation curves were obtained by plotting normalized conductance (G/G_max_) against test pulse voltage (V) and then fitting with Boltzmann functions in the forms of G/G_max_ = 1/{1 + exp[(V_1/2act_ − V)/k]}, where G_max_ is the maximal K^+^ conductance, V_1/2act_ is the voltage for half-maximum activation and k is the slope factor.

For the analysis of steady-state inactivation of I_A_, peak inward currents (I) obtained from steady-state inactivation protocol were normalized to the maximal peak current (I_max_) and fitted with a negative Boltzmann function of the form: I/I_max_ = 1/{1 + exp[(V − V_1/2 inact_)/k]}, where V represents the inactivation prepulse potential, V_1/2 inact_ represents the voltage at which activation is half-maximal and k is the slope factor.

Data were presented as mean ± s.e.m. Statistical analyses were performed using a student’s t-test, one-way or two-way ANOVA with repeated measures followed by Bonferroni adjustments for pairwise comparisons or Tukey’s post hoc test as appropriate. Comparisons of proportions were made using Fisher’s exact test. Significance was set at p < 0.05. The type of statistical test used for each comparison is indicated in the figure legend.

## Additional Information

**How to cite this article**: Qu, L. and Caterina, M. J. Enhanced excitability and suppression of A-type K^+^ currents in joint sensory neurons in a murine model of antigen-induced arthritis. *Sci. Rep*. **6**, 28899; doi: 10.1038/srep28899 (2016).
